# Optical Torque Modulation of Cs_2_AgBiBr_6_ Perovskite-Coated Gold Nanospheres by Vector Bessel Beams

**DOI:** 10.3390/mi17070865

**Published:** 2026-07-21

**Authors:** Ping Li, Chen Yan, Liangchen Lu, Haoyu Wang, Wenxuan Shi, Yiping Han

**Affiliations:** 1 School of Physics, Xidian University, Xi’an 710071, China; 23201110761@stu.xidian.edu.cn (P.L.); 22051110092@stu.xidian.edu.cn (C.Y.); lchengl11@163.com (L.L.); 24201111373@stu.xidian.edu.cn (H.W.); yphan@xidian.edu.cn (Y.H.); 2 School of Aerospace Science and Technology, Xidian University, Xi’an 710071, China

**Keywords:** optical torque, perovskite-coated gold nanospheres, generalized Lorenz–Mie theory, Maxwell stress tensor, multipole scattering

## Abstract

Based on generalized Lorenz–Mie theory (GLMT) and the Maxwell stress tensor (MST) method, this study investigates the modulation mechanism of the axial optical torque *N_z_* exerted on Cs_2_AgBiBr_6_ (CABB) perovskite-coated gold nanospheres under vector Bessel-beam illumination. The results show that the CABB shell reconstructs the torque-resonance channels of the coated particle by modifying both the dispersive dielectric environment around the gold core and the core–shell interfacial response. As the shell thickness increases, the dominant response undergoes a continuous redshift. The polarization state, half-cone angle *α*_0_, and order *l* of the incident vector Bessel beam serve as external optical-field degrees of freedom that regulate the incident angular-momentum channels, thereby enabling coordinated control over the torque peak magnitude, spectral line shape, and torque direction. Analyses of the near-field distributions, Poynting-vector distributions, and Mie-order decomposition reveal that the strong torque response arises from selective coupling between the intrinsic Mie channels of the core–shell particle and the vectorial structure of the incident light, rather than simply from local field-intensity enhancement. This study provides a theoretical basis for tunable *N_z_* responses in perovskite–plasmonic hybrid nanostructures and for structured-light-driven rotational manipulation at the nanoscale.

## 1. Introduction

The exchange of momentum and angular momentum between optical fields and micro-/nanoparticles constitutes the physical basis of optical manipulation [1,2]. Since Ashkin et al. demonstrated a single-beam gradient-force optical trap [3,4], optical tweezers [5] have evolved from their initial application in particle trapping to a broad range of fields, including micro- and nanomanipulation, bioanalysis, single-particle measurements, and mechanical characterization of complex media [6,7,8]. In these studies, optical force [9] primarily reflects the transfer of linear momentum from the optical field to the particle and determines the particle’s trapping, pushing, and transport behavior. As optical manipulation has gradually expanded from position control to orientation and rotational control, optical torque [10,11,12] has attracted increasing attention. Optical torque originates from the transfer of angular momentum from the optical field to the particle and can drive directed particle rotation, thereby providing a rotational degree of freedom independent of translational manipulation for optical wrenches, micro-/nanorotors, and optically driven nanomachines [13,14,15]. Therefore, how to enhance, select, and regulate the transfer of angular momentum from optical fields to particles at the nanoscale has become an important issue in optical manipulation and nanorotor design.

Optical torque regulation can generally be approached from two perspectives: the incident optical field and the particle’s intrinsic response. On the one hand, the angular-momentum input channels can be modified by tailoring the incident optical field [16]. Circularly polarized light carries spin angular momentum [17], while structured optical fields such as vortex beams [18], higher-order Bessel beams [19], and Laguerre–Gaussian beams [20] can carry orbital angular momentum. Among these structured beams, vector Bessel beams are particularly useful because they combine nondiffracting-like propagation with controllable polarization and orbital angular-momentum channels. Their half-cone angle, beam order, and polarization state can independently regulate the spatial spectrum, azimuthal phase structure, and spin-angular-momentum input of the incident field. Therefore, vector Bessel beams provide flexible optical degrees of freedom for selectively coupling to the multipolar scattering channels of nanoparticles. Therefore, the polarization state, topological charge, and spatial spectrum of the incident light can all affect the rotational response of particles [21,22]. On the other hand, the multipolar scattering channels of particles can be modified by engineering their materials and structures [23]. The absorption loss [24], particle geometry [25], material anisotropy, and internal structure [26] can alter the amplitude and phase relationships among different multipolar channels, thereby affecting absorption, scattering, and angular-momentum redistribution. Previous studies have shown that eccentric core–shell nanoparticles [27,28], charged spheres [29,30], PEMC spheres [31,32,33,34], and graphene-coated gold nanospheres [35,36] can exhibit pronounced optical force and optical torque characteristics in specific structured optical fields. These results indicate that optical torque is governed by the combined effects of the spin/orbital angular-momentum input of the incident optical field, the material loss of the particle, and the selective coupling among multipole scattering channels.

Recent studies have further demonstrated that optical torque can be actively controlled by tailoring the spatial phase, polarization, and angular-momentum structure of the incident light. For example, phase-gradient optical fields have been used to realize switchable optical torque and reversible rotation of nanoparticle-assembled micromotors [37]. Superhybrid modes in Mie-resonant particles have also been shown to enhance optical torque through resonant scattering-channel coupling [38]. More recently, single-beam schemes have enabled time-varying three-dimensional optical torque by controlling the transfer of optical angular momentum [12]. These advances indicate that optical torque is not determined solely by the total incident power or local field intensity, but can be selectively regulated by matching structured-light angular-momentum channels to the intrinsic electromagnetic modes of the target particle.

Gold nanospheres [39,40,41,42] are typical objects for studying plasmonic optical manipulation and angular-momentum transfer at the nanoscale. Owing to localized surface plasmon resonance, gold nanoparticles exhibit strong scattering, absorption, and near-field enhancement in the visible spectral range. Their resonant position, modal phase, and local energy-flow distribution are highly sensitive to particle size, the surrounding dielectric environment, and surface-modification layers [43,44,45]. To further tune the resonance position and modal response of gold nanoparticles, core–shell structures have been widely used in the optical design of composite nanoparticles [46,47]. For optical torque, the role of the shell is not limited to changing the effective particle size; it may also affect the efficiency of net axial angular-momentum transfer by regulating the dielectric environment around the gold core, absorption loss, and the phase relationships among different Mie channels. Therefore, coated gold nanospheres provide a representative model for studying the coupling among the material shell, multipolar scattering channels, and optical angular-momentum input.

Previous studies on graphene-coated gold nanospheres [48] have shown that surface-conducting coatings can significantly affect the optical force and torque responses under vector Bessel-beam illumination. However, graphene coatings primarily govern the electromagnetic boundary conditions via surface conductivity [49]. In contrast, a finite-thickness perovskite [50] shell can serve as a dispersive and absorptive dielectric environment surrounding the gold core. Such a shell can continuously modify the effective electromagnetic environment, the core–shell interfacial response, and the Mie scattering channels of the whole particle. This mechanism is different from those of bare metallic nanoparticles, eccentric core–shell particles, and surface-conducting graphene-coated systems, which mainly rely on intrinsic plasmonic resonance, geometrical symmetry breaking, or surface-conductivity-induced boundary modulation. Therefore, the novelty of the present structure lies not only in replacing the coating material but also in introducing a finite-thickness, dispersive, and absorptive perovskite shell to reconstruct the plasmonic Mie channels responsible for axial angular-momentum transfer. The advantage of the core–shell Cs_2_AgBiBr_6_ perovskite-coated gold nanosphere lies in its ability to combine the strong plasmonic response of the Au core with the dispersive and absorptive modulation capability of the perovskite shell. Compared with bare Au nanoparticles, the perovskite shell provides an additional structural degree of freedom to tune the resonance wavelength and torque magnitude without altering the Au core. Compared with conventional low-index dielectric shells, the Cs_2_AgBiBr_6_ shell introduces stronger visible-range dispersion and absorption-assisted coupling. Compared with graphene-coated gold nanospheres, the finite-thickness perovskite shell can continuously modify the effective dielectric environment and the core–shell interfacial response, rather than merely changing the surface-conductivity boundary condition. Therefore, this hybrid structure provides a more flexible platform for regulating axial optical torque through the combined modulation of plasmonic resonance, shell-induced dispersion, absorption loss, and angular-momentum-channel matching.

Perovskite shells [51,52] provide a distinct route, distinct from conventional dielectric coatings, for regulating the plasmonic response and angular-momentum transfer of gold nanospheres [53]. Compared with low-refractive-index dielectric shells, perovskite materials usually possess a relatively high refractive index, pronounced dispersion, and certain absorption, which can effectively modify the effective electromagnetic environment around the gold core and further influence the multipolar resonance channels of core–shell particles [54,55,56]. Cs_2_AgBiBr_6_ (CABB), as a lead-free double perovskite material, possesses a relatively high refractive index, visible-range dispersion, and non-negligible absorption loss [57,58]. These optical characteristics make CABB suitable as a functional coating layer to modify the local dielectric environment and the plasmonic response of gold nanostructures. Recent studies on perovskite–plasmonic hybrid systems have shown that the coupling between metal nanoparticles and Cs_2_AgBiBr_6_-based materials can effectively enhance light harvesting and regulate electromagnetic responses, indicating the potential of CABB-related structures for tunable nanophotonic applications [51,59]. Compared with conventional low-index dielectric shells, a CABB shell can introduce stronger dispersive modulation and absorption-assisted coupling in the visible spectral range. Compared with graphene-coated gold nanospheres, in which the coating primarily modifies the electromagnetic boundary conditions via surface conductivity, a finite-thickness CABB shell can continuously tune the effective dielectric environment around the gold core and the core–shell interfacial response. Therefore, CABB-coated gold nanospheres provide a distinct platform for regulating the plasmonic scattering channels and optical angular-momentum transfer of core–shell nanoparticles.

Previous studies have shown that a CABB shell can regulate the axial optical force *F_z_* exerted on gold nanospheres under polarized Bessel-beam illumination and induce shifts in the optical-force resonance as the shell thickness varies [60]. However, *F_z_* mainly describes linear-momentum transfer along the propagation direction, and its variation cannot directly predict the magnitude or sign of the axial optical torque. The axial optical torque *N_z_* corresponds to optical angular-momentum transfer to the particle and depends not only on the particle resonance, but also on the incident polarization state, Bessel-beam order *l*, azimuthal angular-momentum channels, and phase relationships among the Mie scattering coefficients. Therefore, whether a CABB shell can convert its modulation of gold-core scattering resonances into controllable axial angular-momentum transfer, thereby enabling regulation of the torque peak position, magnitude, spectral line shape, and direction, remains an issue that requires further clarification.

Motivated by the above issues, this study systematically investigates the axial optical torque *N_z_* exerted on CABB perovskite-coated gold nanospheres under vector Bessel-beam illumination. The focus is placed on the joint modulation of *N_z_* by the Cs_2_AgBiBr_6_ shell and the characteristics of the structured optical beam, with the aim of revealing the matching mechanism between the core–shell Mie channels and the angular-momentum channels of the incident vector optical field. Such a controllable axial torque response may be useful for tunable optical nanorotors, reversible rotational manipulation of nanoparticles, polarization-selective optical manipulation, and structured-light-driven perovskite–plasmonic optomechanical devices. On this basis, we further analyze how the material shell, core–shell interfacial response, and angular-momentum input of the structured light jointly determine the peak position, magnitude, and sign of *N_z_*. The remainder of this paper is organized as follows. Section 2 presents the theoretical model, including the vector Bessel-beam expansion, the Mie scattering coefficients of CABB-coated gold nanospheres, and the calculation of *N_z_* based on the Maxwell stress tensor. Section 3 presents the numerical results and discussion. First, the torque spectra of different particle structures are compared to analyze the modulation of the peak position and peak magnitude by the CABB shell. The effects of shell thickness, gold-core radius, polarization state, half-cone angle, and Bessel-beam order on *N_z_* are then discussed. Finally, the physical origin of the strong torque response is explained by combining the near-field distributions, energy-flow distributions, and Mie-order decomposition. Section 4 summarizes the main conclusions of this work.

## 2. Theoretical Model

### 2.1. Geometry and Material Parameters

**Figure 1:**
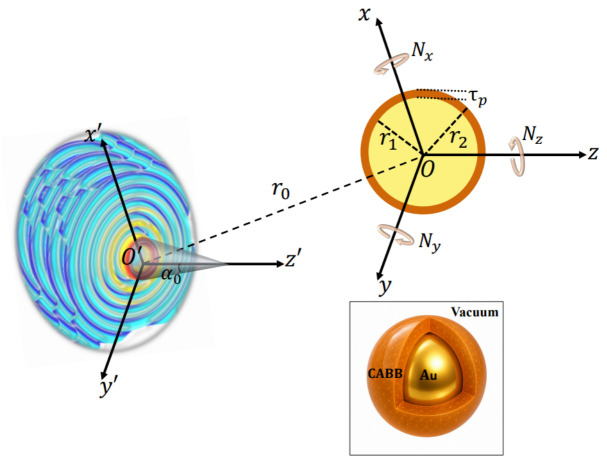
Schematic geometry of a CABB perovskite-coated gold nanosphere under illumination by a structured-light field, with a Bessel beam used as an example in this study. The gold-core radius $r_{1}$, outer radius $r_{2}$, shell thickness $\tau_{p}=r_{2}-r_{1}$, and half-cone angle $\alpha_{0}$ of the incident beam are indicated. The inset shows the relative arrangement of the constituent materials.

This study considers a concentric core–shell nanosphere consisting of a gold nanosphere core with radius $r_{2}$ and refractive index $m_{1}$, surrounded by a CABB perovskite shell with refractive index $m_{2}$. The total radius of the coated sphere is $r_{2}=r_{1}+\tau_{p}$, where $\tau_{p}$ denotes the shell thickness. The particle is embedded in a homogeneous, nonmagnetic background medium with refractive index $m_{3}$, which is taken as $m_{3}=1$ unless otherwise specified, and its center is located at the origin O of the O-xyz coordinate system. The particle is illuminated by a Bessel beam propagating along the z-direction, defined in the O’-x’y’z’ beam coordinate system, where O’ denotes the beam center, and $\alpha_{0}$ is the half-cone angle, as shown in Figure 1:

The dielectric response of the gold core is described using a local Drude–Sommerfeld model with a phenomenological size-dependent electron-collision rate, as given in Equations (1) and (2). This model includes the bulk electron-collision contribution and an additional surface-scattering correction and is adopted here as an effective classical description of the gold-core dielectric response [61]. The absorption loss of the Au core is included through the imaginary part of the complex dielectric function $\varepsilon_{\mathrm{Au}}(\lambda )$. Physically, Im[εAu] represents loss in the metal, corresponding to the conversion of part of the incident electromagnetic energy into internal energy of the nanoparticle. According to the electromagnetic energy dissipation relation derived from Poynting’s theorem [62,63], this energy loss can be evaluated from the volume integral of the loss density inside the Au core. Equivalently, within the Mie framework, it can be characterized by the absorption cross section $C_{\mathrm{abs}}$. In the present GLMT-MST calculation, the absorption loss is incorporated into the complex Mie scattering coefficients, thereby affecting the resonance linewidth, torque magnitude, and angular-momentum transfer efficiency. However, it must be emphasized that this work focuses on the electromagnetic optical torque. The absorption-induced heating, heat conduction, and thermo-optic feedback are not included.

(1) $$\varepsilon_{\mathrm{Au}}(\omega )=\varepsilon_{b}-\frac{\omega_{\mathrm{Au}}^{2}}{\omega \left(\omega +i\gamma_{\mathrm{Au}}\right)}$$

where

(2) $$\gamma_{\mathrm{Au}}=\gamma_{\mathrm{bulk}}+\frac{Av_{F}}{a_{\mathrm{eff}}}$$

Here ω is the angular frequency, $\varepsilon_{b}$ is the background dielectric constant, $\omega_{\mathrm{Au}}$ is the plasma frequency of gold, $\gamma_{\mathrm{Au}}$ is the size-dependent damping frequency of gold, $\gamma_{\mathrm{bulk}}$ is the electron-collision frequency, *A* is an empirical correction coefficient used to match the theoretical model with experimental results, $v_{F}$ is the Fermi velocity of electrons, and $a_{\mathrm{eff}}$ is the effective radius of the particle. The refractive index of the Cs_2_AgBiBr_6_ shell is written as $m_{2}(\lambda )=n_{2}(\lambda )+i\kappa_{2}(\lambda )$, where $n_{2}(\lambda )$ and $\kappa_{2}(\lambda )$ denote the dispersive dielectric response and absorption loss, respectively. In this work, the optical constants of Cs_2_AgBiBr_6_ are taken from reported experimental optical data of Cs_2_AgBiBr_6_ thin films. In particular, the complex refractive-index data (n+iκ) in the wavelength range of 0.300–1.70 μm have been reported for Cs_2_AgBiBr_6_ thin films at room temperature and are available in the refractiveindex.info optical-constants database [57,64]. Therefore, both the visible-range dispersion and absorption loss of the Cs_2_AgBiBr_6_ shell are included in the GLMT calculation through the complex refractive index. The real part $n_{2}(\lambda )$ modifies the effective dielectric environment around the gold core and shifts the core–shell Mie resonance channels, whereas the imaginary part $\kappa_{2}(\lambda )$ accounts for absorption loss in the perovskite shell and affects the torque magnitude and spectral linewidth. In the present study, we focus on the electromagnetic contribution to the optical torque. Absorption-induced temperature rise, heat conduction, and thermo-optic feedback are not included and will be considered in future work. In this study, the electromagnetic optical torque response is investigated under fixed material parameters, without further coupling to absorption-induced temperature rise, heat conduction, or thermo-optic feedback. Therefore, the parameters are taken as follows: $\varepsilon_{b}=9.8$, $\omega_{\mathrm{Au}}=9\;\mathrm{eV}$, $\gamma_{\mathrm{bulk}}=0.066\;\mathrm{eV}$, A=0.25, and $v_{F}=1.4\times 10^{6}\;m/s$.

However, it should be noted that the damping rate in Equation (2) contains only the bulk-collision and surface-scattering contributions. It does not explicitly include the additional plasmon damping and dielectric-response renormalization associated with Lorentz friction, namely the radiation-reaction effect arising from the collective oscillation of conduction electrons in a metallic nanoparticle. Although the electromagnetic radiation of the entire particle is included at the field level through the outgoing-wave Mie solution, the corresponding nanoscale modification of the intrinsic dielectric response of gold is not incorporated into Equation (1). Recent studies based on the random phase approximation have shown that Lorentz-friction and nonharmonic effects may become significant for gold nanoparticles with radii of several tens of nanometers, potentially modifying their resonance positions, linewidths, and optical responses. Therefore, the dielectric model adopted in this work should be regarded as an effective classical local-response approximation. For small gold cores, particularly r1 = 10 nm, this approximation remains reasonably reliable. However, the results for larger gold cores should be interpreted with caution because nonlocal and nonharmonic effects are not included in the present dielectric model [65].

### 2.2. Incident-Field Expansion and Scattering by the Core–Shell Sphere

Within the framework of generalized Lorenz–Mie theory (GLMT), an arbitrary incident optical field can be expanded as a linear combination of vector spherical wave functions. For a structured optical field propagating along the z-direction, the incident electric and magnetic fields can be written as [66]:

(3) $$E_{i}=\sum_{n=1}^{\infty }\sum_{m=-n}^{n}c_{n}^{pw}\left[g_{n,TM}^{m,u}N_{mn}^{\left(1\right)}(kr)+ig_{n,TE}^{m,u}M_{mn}^{\left(1\right)}(kr)\right]$$

(4) $$H_{i}=-\frac{ik}{\omega }\sum_{n=1}^{\infty }\sum_{m=-n}^{n}c_{n}^{pw}\left[g_{n,TM}^{m,u}M_{mn}^{\left(1\right)}(kr)+ig_{n,TE}^{m,u}N_{mn}^{\left(1\right)}(kr)\right]$$

where $k_{3}=\frac{2\pi m_{3}}{\lambda }$ is the wavenumber in the background medium, *n* denotes the Mie multipole order, *m* labels the azimuthal angular-momentum channel, and $c_{n}^{pw}$ is defined as $c_{n}^{pw}=\left\{\begin{array}{ll} i^{n+1}\frac{2n+1}{n(n+1)} & m\geq 0\\ i^{n+1}{\left(-1\right)}^{m}\frac{(n-m)!}{(n+m)!}\frac{2n+1}{n(n+1)} & m<0 \end{array}\right.$. Here, $g_{mn}^{\mathrm{TM}}$ and $g_{mn}^{\mathrm{TE}}$ denote the beam shape coefficients (BSCs) associated with the TM and TE components of the incident field, respectively, while $M_{mn}^{\left(1\right)}$ and $N_{mn}^{\left(1\right)}$ are vector spherical wave functions of the first kind. The spatial distribution, polarization state, and propagation characteristics of different structured-light fields are all represented by the BSCs. In this work, vector Bessel beams are used as representative incident fields, whose BSCs are determined jointly by the beam order *l*, the half-cone angle $\alpha_{0}$, the polarization state, and the beam-center position, as given in Appendix A. In this study, six typical incident cases are considered, including *x*-linear polarization (*xp*), *y*-linear polarization (*yp*), left-handed circular polarization (*lc*), right-handed circular polarization (*rc*), radial polarization (*rp*), and azimuthal polarization (*ap*), to analyze the effects of different angular-momentum input channels on the *N_z_* of the core–shell particle.

Similar to the incident field, the scattered electric and magnetic fields in the exterior region of the CABB-coated gold nanosphere can be expressed as [66]:

(5) $$E_{s}=\sum_{n=1}^{\infty }\sum_{m=-n}^{n}c_{n}^{pw}\left[A_{n}^{m,u}N_{mn}^{\left(4\right)}(kr)+iB_{n}^{m,u}M_{mn}^{\left(4\right)}(kr)\right]$$

(6) $$H_{s}=-\frac{ik}{\omega }\sum_{n=1}^{\infty }\sum_{m=-n}^{n}c_{n}^{pw}\left[A_{n}^{m,u}M_{mn}^{\left(4\right)}(kr)+iB_{n}^{m,u}N_{mn}^{\left(4\right)}(kr)\right]$$

Here, $M_{mn}^{\left(4\right)}$ and $N_{mn}^{\left(4\right)}$ denote the fourth-kind vector spherical wave functions satisfying the outgoing radiation condition, while $A_{n}^{m,u}$ and $B_{n}^{m,u}$ are the scattering-field coefficients jointly determined by the incident-field BSCs and the conventional Mie coefficients $a_{n}$ and $b_{n}$. The explicit expressions for these coefficients are listed in Appendix B.

### 2.3. Optical Torque Based on the Maxwell Stress Tensor

Once the incident and scattered fields have been determined, the total field in the exterior region of the particle is given by their superposition:

(7) $$E=E_{i}+E_{s},\;H=H_{i}+H_{s}$$

The axial optical torque considered here originates from the transfer of optical angular momentum from the incident vector Bessel beam to the core–shell nanosphere. A vector Bessel beam can carry spin angular momentum through its polarization state and orbital angular momentum through its azimuthal phase structure. In particular, circular polarization contributes spin angular momentum, while a nonzero Bessel-beam order *l* introduces an azimuthal phase factor, thereby providing an orbital angular-momentum channel. When the beam interacts with an absorptive and scattering core–shell particle, the incident angular-momentum flux is redistributed through absorption and scattering. The imbalance between the incoming and outgoing angular-momentum fluxes produces a net axial optical torque *N_z_* on the particle. In the GLMT-MST formulation, this process is described by the coupling between the beam shape coefficients of the incident vector Bessel beam and the Mie scattering coefficients of the coated nanosphere.

After the total field in the exterior region is determined, the optical torque exerted on the particle is obtained by integrating the Maxwell stress tensor over a closed surface *S* surrounding the particle [62]:

(8) $$\langle N\rangle =\langle \oint_{S}r\times (T\cdot \hat{n})dS\rangle$$

where r denotes the position vector on the integration surface, $\hat{n}$ is the outward unit normal to the surface, *T* is the Maxwell stress tensor (MST), and 〈 〉 represents time averaging. In a homogeneous nonmagnetic background medium, the time-averaged Maxwell stress tensor is given by [63]:

(9) T=12Reε3EE*+μ3HH*−12ε3|E|2+μ3|H|2I

where $\varepsilon_{3}$ and $\;\mu_{3}$ denote the permittivity and permeability of the background medium, respectively, *I* is the identity tensor, and the superscript ∗ denotes complex conjugation. By substituting Equations (7) and (9) into Equation (8), the analytical expression for the optical torque acting on the core–shell nanosphere is obtained:

(10) $$\begin{array}{l} T_{x}^{u}=\frac{4m_{3}}{c}\frac{\pi }{k^{3}}\sum_{n=1}^{\infty }\sum_{m=1}^{n}C_{n}^{m}ℜ\left(A_{n}^{m,u}\right)\\ T_{y}^{u}=\frac{4m_{3}}{c}\frac{\pi }{k^{3}}\sum_{n=1}^{\infty }\sum_{m=1}^{n}C_{n}^{m}ℑ\left(A_{n}^{m,u}\right)\\ T_{z}^{u}=-\frac{4m_{3}}{c}\frac{\pi }{k^{3}}\sum_{n=1}^{\infty }\sum_{m=1}^{n}mC_{n}^{m}B_{n}^{m,u} \end{array}$$

where

(11) $$\begin{array}{l} C_{n}^{m}=\frac{2n+1}{n(n+1)}\frac{(n+|m|)!}{(n-|m|)!}\\ A_{n}^{m,u}=A_{n}\left(g_{n,TM}^{m-1,u}g_{n,TM}^{m,u*}-g_{n,TM}^{-m,u}g_{n,TM}^{-m+1,u*}\right)+B_{n}\left(g_{n,TE}^{m-1,u}g_{n,TE}^{m,u*}-g_{n,TE}^{-m,u}g_{n,TE}^{-m+1,u*}\right)\\ B_{n}^{m,u}=A_{n}\left({\left|g_{n,TM}^{m,u}\right|}^{2}-{\left|g_{n,TM}^{-m,u}\right|}^{2}\right)+B_{n}\left({\left|g_{n,TE}^{m,u}\right|}^{2}-{\left|g_{n,TE}^{-m,u}\right|}^{2}\right)\\ A_{n}=ℜ\left(a_{n}\right)-{\left|a_{n}\right|}^{2}\\ B_{n}=ℜ\left(b_{n}\right)-{\left|b_{n}\right|}^{2} \end{array}$$

**Figure 2:**
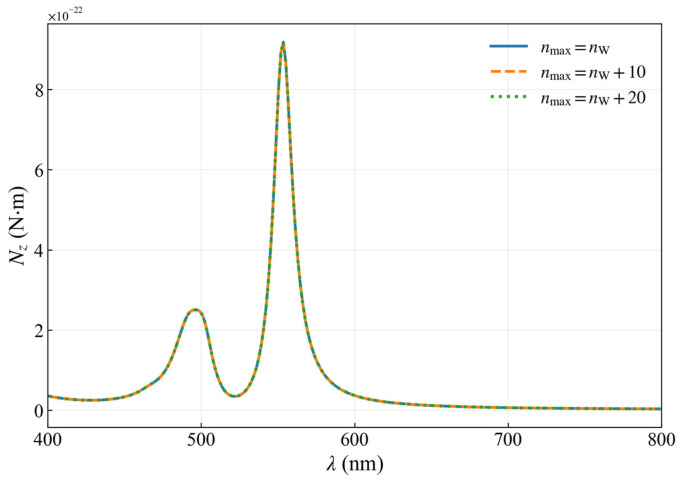
Convergence test of the axial optical torque spectrum for different multipole truncation orders. The curves correspond to $n_{W}=5$, $n_{W}=15$, and $n_{W}=25$. The calculation parameters are *r*_1_ = 10 nm, $\tau_{p}$ = 4 nm, *l* = 1, and *α*_0_ = 30°, under radially polarized vector Bessel-beam illumination.

In the numerical implementation, the infinite multipole series in the GLMT-MST formulation was truncated at a finite maximum multipole order $n_{\max}$. The initial truncation order $n_{W}$ was estimated using the Wiscombe criterion [67], $n_{W}=\left[x+4x^{1/3}+2\right]$, where $x=kx_{2}$ is the size parameter of the whole coated sphere, and [ ] denotes rounding up to the nearest integer. To further ensure convergence, a correction term *d_n_* was introduced, and the actual truncation order was taken as $n_{\max}=n_{W}+d_{n}$. Representative calculations of torque with *d_n_* = 0, 10, and 20 are shown in Figure 2, where the calculation parameters are $r_{1}=10$ nm, $\tau_{p}=4$ nm, *l* = 1, and *α*_0_ = 30°, under radially polarized vector Bessel-beam illumination. Therefore, $n_{W}=5$ based on the Wiscombe criterion. The results show that increasing *d_n_* produces negligible changes in the peak position, spectral line shape, and peak magnitude of the calculated axial optical torque *N_z_*. Therefore, the higher-order multipole contributions beyond the adopted truncation order have little influence on the final torque spectra for the particle sizes considered in this work. This confirms the numerical convergence and computational efficiency of the present GLMT-MST calculation.

## 3. Numerical Results and Discussions

Based on the GLMT–MST theoretical model established in Section 2, this section further analyzes the axial optical torque *N_z_* exerted on CABB-coated gold nanospheres under vector Bessel-beam illumination. As indicated by Equations (10) and (11), *N_z_* is jointly determined by the beam shape coefficients of the incident optical field, the Mie scattering coefficients of the core–shell sphere, and the coherent combination among different azimuthal channels. Therefore, this section’s discussion focuses on three questions. First, whether the CABB shell can reconstruct the torque-resonance channels in a manner distinct from a simple size effect. Second, how the shell thickness and gold-core size modify the intrinsic scattering response of the core–shell particle. Third, how the polarization state, half-cone angle, and order of the vector Bessel beam regulate the incident angular-momentum channels and further affect the magnitude, spectral line shape, and direction of *N_z_*.

### 3.1. Effect of Core–Shell Configuration on N_z_

**Figure 3:**
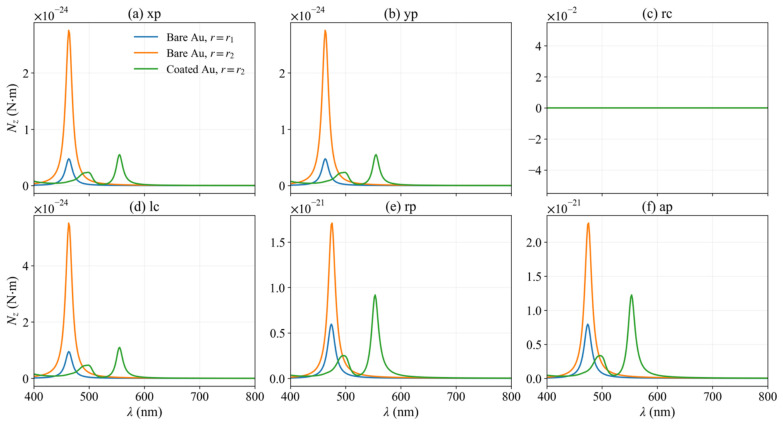
Comparison of axial optical torque spectra for a bare gold core, a bare gold sphere with the same outer radius, and a CABB perovskite-coated gold sphere under different polarization states. The calculation parameters are a gold-core radius *r*_1_ = 10 nm, shell thickness $\tau_{p}$ = 4 nm, Bessel-beam order *l* = 1, and half-cone angle *α*_0_ = 30°.

To distinguish the material effect of the CABB shell from the geometrical effect caused by the increase in the particle outer radius, Figure 3 compares the *N_z_* spectra of a bare gold core (*r* = *r*_1_), a bare gold sphere with the same outer radius (*r* = *r*_2_), and a CABB perovskite-coated gold sphere (*r* = *r*_2_) under the same vector Bessel-beam conditions. Here, the bare gold sphere with the same outer radius can be regarded as a solid gold sphere obtained by replacing the CABB shell with a gold layer of the same thickness. The calculation parameters are a bare gold-core radius of (*r*_1_ = 10 nm), shell thickness of ($\tau_{p}$ = 4 nm), Bessel-beam order of *l* = 1, and half-cone angle of (*α*_0_ = 30°).

As shown in Figure 3, the main torque responses of the bare gold core and the bare gold sphere with the same outer radius are concentrated in the short-wavelength region. In contrast, the CABB-coated structure exhibits different degrees of redshift under *xp*, *yp*, *lc*, *rp*, and *ap* polarizations, with new torque-resonance peaks appearing in the wavelength range of approximately 500–560 nm. It should be noted that the peak positions of the CABB-coated structure do not coincide with those of the bare gold sphere with the same outer radius. This indicates that the spectral shifts cannot be simply attributed to an increase in the particle outer radius, but mainly originate from the modulation of the effective electromagnetic environment around the gold core, the core–shell interfacial response, and the Mie scattering coefficients of the coated sphere by the CABB shell. According to the expression for *N_z_* given in Section 2, namely Equations (10) and (11), the axial torque is jointly determined by the Mie coefficients and the beam shape coefficients. Therefore, when the incident-light parameters are kept unchanged, the changes in the resonance peak positions and amplitudes in Figure 3 mainly reflect the reconstruction of the intrinsic scattering response of the particle and the axial angular-momentum transfer channels induced by the CABB shell. In addition, *N_z_* remains close to zero for all structures under *rc* polarization, indicating that, under the present conditions of *l* = 1, *α*_0_ = 30°, and axisymmetric geometry, the contributions of the relevant azimuthal channels to the net axial torque are strongly suppressed. These results show that the formation of a strong *N_z_* depends not only on the resonance response of the core–shell particle, but also on effective matching between the particle scattering channels and the angular-momentum channels of the incident vector optical field.

### 3.2. Effect of CABB Shell Thickness on N_z_

**Figure 4:**
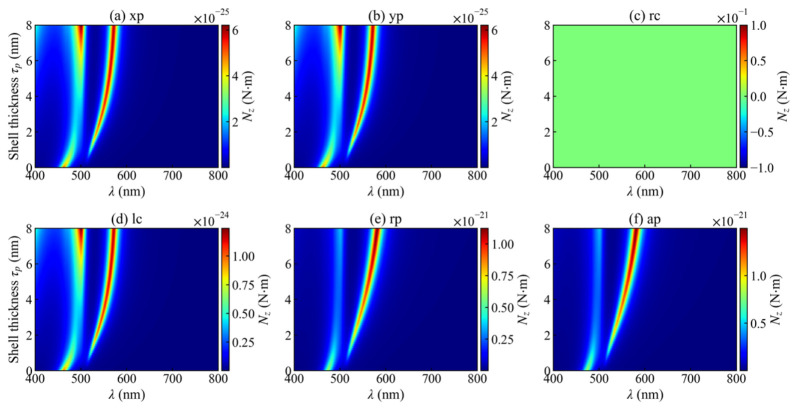
Two-dimensional distributions of the axial optical torque under modulation by the CABB shell thickness. The horizontal axis represents the incident wavelength, and the vertical axis represents the shell thickness. The calculation parameters are a gold-core radius *r*_1_ = 10 nm, Bessel-beam order *l* = 1, and half-cone angle *α*_0_ = 30°.

After confirming that the CABB shell can participate in torque modulation in a manner distinct from a simple size effect, it is necessary to further analyze the continuous modulation of *N_z_* by the shell thickness $\tau_{p}$. Figure 4 shows the two-dimensional distributions of *N_z_* as functions of the incident wavelength and CABB shell thickness for *r*_1_ = 10 nm, *l* = 1, and *α*_0_ = 30°.

As shown in Figure 4 the two-dimensional torque distributions under *xp*- and *yp*-polarized incidence are nearly identical. This indicates that the concentric core–shell sphere exhibits approximately equivalent axial torque responses to the two orthogonal linear polarizations. Two main response branches can be observed. These correspond to the short-wavelength and long-wavelength branches of the torque-resonance peaks. As the coating thickness increases, the short-wavelength branch gradually shifts from around 450 nm to approximately 500 nm. The long-wavelength branch redshifts from about 520 nm to approximately 580–600 nm. Similarly, the torque obtained under *lc*-polarized incidence also exhibits a comparable two-branch structure, but with an overall magnitude higher than that under linearly polarized incidence. In contrast, under *rp*- and *ap*-polarized incidence, the strong torque responses are mainly concentrated along the long-wave.length branch, with magnitudes reaching the order of 10^−21^ N·m, which are significantly higher than those under linear and circular polarizations. These results indicate that the CABB shell thickness not only affects the peak magnitude of *N_z_*, but also induces a continuous redshift in the wavelength corresponding to the torque-resonance peak. This suggests that the shell thickness can serve as a structural degree of freedom for regulating the intrinsic scattering response of the core–shell particle and the efficiency of axial angular-momentum transfer. Meanwhile, differences in torque magnitude and response branches across different polarization states also indicate that the shell-mediated modulation must be matched to the vectorial structure of the incident light to generate a strong axial torque.

**Figure 5:**
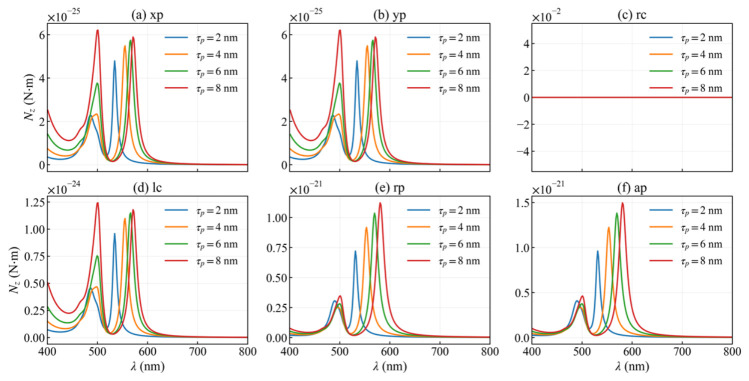
Axial optical torque spectra of coated gold nanospheres with different CABB shell thicknesses. The variations in *N_z_* with incident wavelength are shown for $\tau_{p}$ = 2, 4, 6, and 8 nm. Other parameters are *r*_1_ = 10 nm, *l* = 1, and *α*_0_ = 30°.

To further verify the spectral evolution induced by the shell thickness observed in Figure 4, Figure 5 presents representative *N_z_* spectra for $\tau_{p}$ = 2, 4, 6, and 8 nm, with the other parameters kept the same as those used in Figure 4. A comparison between Figure 5a,b shows that, under *xp*- and *yp*-polarized incidence, the two spectra almost completely overlap, further confirming that the concentric core–shell structure exhibits approximately equivalent torque responses to the two orthogonal linear polarizations. As the CABB shell thickness increases, the torque spectra do not simply undergo an overall enhancement, but instead exhibit pronounced peak shifts and spectral-profile reconstruction. The short-wavelength response peak is mainly located within the range of 480–510 nm, whereas the long-wavelength response peak gradually shifts from around 530 nm to approximately 570 nm as the shell thickness increases. Under *lc*-polarized incidence, the spectral evolution is similar to that observed under linearly polarized incidence, but the peak magnitude is further enhanced. Under *rp*- and *ap*-polarized incidence, the torque responses are mainly concentrated in the long-wavelength region, and the resonance peaks continuously redshift with increasing shell thickness. The torque magnitude is also significantly higher than that under linear and circular polarizations. These results show that modulation of *N_z_* by the CABB shell thickness is not limited to changes in peak magnitude but also affects the resonance wavelength, spectral line shape, and torque magnitude across different polarization states. This phenomenon indicates that the shell thickness is an important parameter for regulating the axial angular-momentum transfer efficiency and operating wavelength of core–shell nanoparticles.

**Figure 6:**
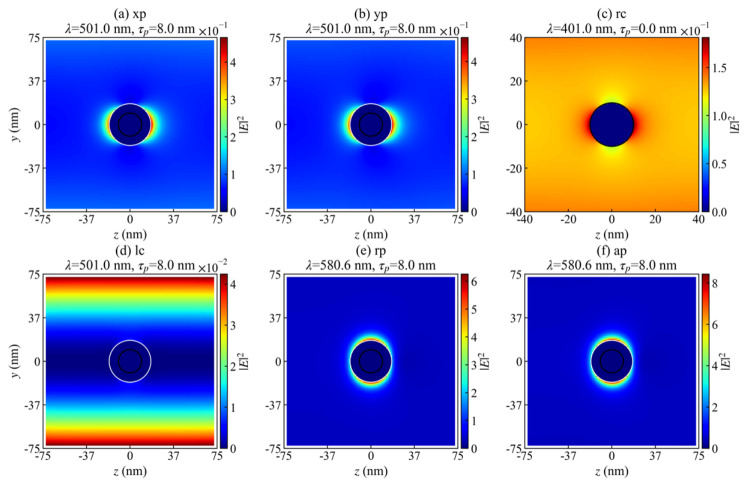
Near-field electric-field-intensity distributions at representative axial torque peak positions selected from Figure 5. The cases of *xp*, *yp*, and *lc* correspond to λ = 501.0 nm and $\tau_{p}$ = 8.0 nm, while those of *rp* and *ap* correspond to λ = 580.6 nm and $\tau_{p}$ = 8.0 nm. The *rc* case is included as a near-zero-torque reference.

**Figure 7:**
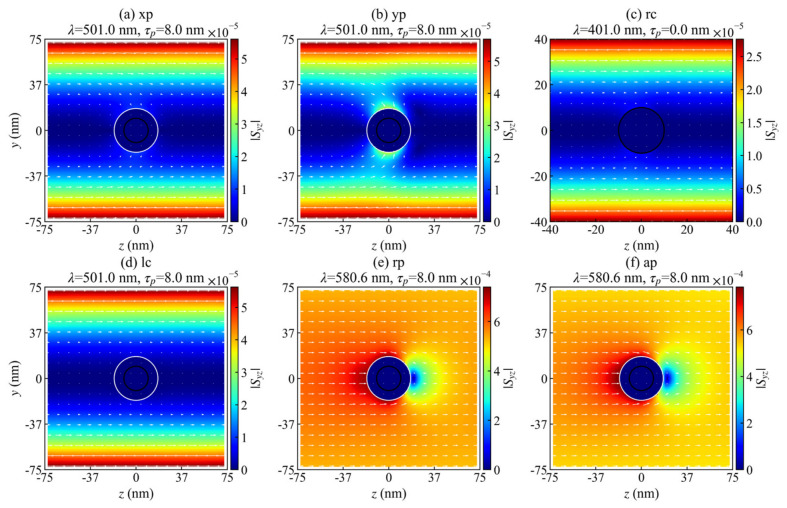
Projected Poynting-vector distributions in the *yz* cross section under the representative wavelength and shell-thickness conditions used in Figure 6. The color map represents the magnitude of the energy flow in the cross section, while the arrows indicate the direction of the energy flow in the *yz* plane. The calculation parameters are the same as those in Figure 6. The outline of the coating is represented in white, the outline of the gold nano-nucleus is represented in black, and the arrows in the figure indicate the propagation direction of the energy flow.

To reveal the variations in the local field and energy flow under shell-thickness modulation, Figure 6 and Figure 7 present the near-field electric-field-intensity distributions and the projected Poynting-vector distributions in the *yz* plane, respectively, at representative response points selected from Figure 5. Specifically, the *xp*-, *yp*-, and *lc*-polarized cases correspond to $\tau_{p}$ = 8.0 nm and λ = 501.0 nm, whereas the *rp*- and *ap*-polarized cases correspond to $\tau_{p}$ = 8.0 nm and λ = 580.6 nm. Since *N_z_* remains close to zero under *rc*-polarized incidence, the case with $\tau_{p}$ = 0 nm and λ = 401.0 nm is selected as a near-zero-torque reference. In the figures, the black contours denote the gold-core boundary, and the white contours denote the CABB shell boundary.

Figure 6 shows that the torque differences under different polarization states are closely related to the local field distributions around the coated sphere. Under *xp*- and *yp*-polarized incidence, the near-field distributions are nearly identical, with strong fields mainly distributed on both sides of the particle along the propagation direction. This is consistent with the nearly overlapping torque spectra shown in Figure 5. Although the *lc*-polarized case is evaluated at the same shell thickness and wavelength as the linearly polarized cases, its field distribution exhibits more pronounced background fringes, and the local enhancement near the particle boundary is relatively more dispersed. This indicates that, even when the variation trends of *N_z_* are similar, the field-coupling mechanisms corresponding to *N_z_* can still differ depending on the polarization mode of the incident light. In contrast, under *rp*- and *ap*-polarized incidence, the strong fields are mainly concentrated near the gold-core–CABB-shell interface and exhibit a more pronounced ring-like enhancement feature, corresponding to the strong torque response on the order of 10^−21^ N·m in Figure 5. Under *rc*-polarized incidence, although near-field perturbations are still present, *N_z_* remains close to zero. This indicates that enhancement of the local electric-field intensity alone cannot determine the net axial torque *N_z_*. The formation of *N_z_* also depends on the angular-momentum flux through the closed integration surface, as well as the coherent superposition and cancelation among different azimuthal scattering channels.

Furthermore, Figure 7 presents the Poynting-vector distributions in the *yz* cross section around the coated sphere under the same parameter conditions, where the color map represents the magnitude of the energy flow in the cross section and the arrows indicate the local propagation direction of the energy flow within that plane. Besides, the outline of the coating is represented in white, the outline of the gold nano-nucleus is represented in black. Under *xp*-, *yp*-, and *lc*-polarized incidence, the energy flow remains distributed mainly along the background propagation direction, with only slight deflection and local compression appearing near the particle. Among these cases, the arrow bending near the particle boundary is slightly more pronounced under *yp*-polarized incidence than under *xp*- and *lc*-polarized incidence. Nevertheless, the overall energy flow is still dominated by the incident propagation direction, indicating that the core–shell particle has a relatively limited effect on the redistribution of local momentum flow under these polarization states. In contrast, under *rp*- and *ap*-polarized incidence, the energy-flow direction near the core–shell boundary undergoes more pronounced bending and circulation. Stronger compression, deflection, and nonuniform distribution of the energy flow appear in the front and rear regions of the particle, while the energy-flow magnitude in the cross section reaches the order of 10^−4^, higher than the 10^−5^ level observed under *xp*-, *yp*-, and *lc*-polarized incidence. This indicates that strong-torque peaks are accompanied not only by field enhancement near the core–shell boundary, but also by more pronounced local momentum-flow deflection and energy-flow redistribution. It should be noted that Figure 7 only shows the projected Poynting-vector distributions in the *yz* cross section. Therefore, the arrow directions can serve only as two-dimensional visual evidence of local momentum-flow redistribution and cannot, by themselves, determine the complete axial optical torque *N_z_*. Strictly speaking, the net axial torque *N_z_* should still be determined by integrating the Maxwell stress tensor over a closed surface enclosing the particle. Taken together, Figure 5, Figure 6 and Figure 7 show that the CABB shell thickness modifies the efficiency of axial angular-momentum transfer from the incident vector Bessel beam to the coated gold nanosphere by regulating the local field enhancement, energy-flow magnitude, and energy-flow direction near the core–shell boundary. Therefore, the thickness-dependent modulation mechanism in this work cannot be ascribed to a simple size effect, but results from the combined action of the material shell, the core–shell interfacial response, and the angular-momentum channels of the structured-light field.

It should be noted that the present GLMT-MST calculation is based on an ideal concentric core–shell sphere with a smooth interface. In realistic samples, material inhomogeneity, local shell-thickness fluctuations, shell roughness, and slight deviations from spherical geometry may occur. These imperfections may influence the optical torque in several ways. First, variations in the optical constants of the Cs_2_AgBiBr_6_ shell may slightly shift the resonance wavelength and alter the torque magnitude, as the Mie coefficients are sensitive to the shell’s complex refractive index. Second, local shell-thickness fluctuations or shell roughness can lead to an inhomogeneous core–shell interfacial response, which may broaden the torque-resonance peaks and reduce the maximum value of *N_z_*. The shell-thickness-dependent results in Figure 4 and Figure 5 also provide an indirect indication of the sensitivity of *N_z_* to thickness variations: changing the shell thickness shifts the resonance wavelength and modifies the peak magnitude, but does not alter the basic polarization-dependent modulation mechanism. Third, deviations from ideal spherical geometry may break the particle’s axial symmetry, induce coupling among additional azimuthal channels, and possibly generate transverse torque components. However, the quantitative treatment of rough, nonspherical, or strongly inhomogeneous particles would require full-wave numerical methods such as the finite-element method, finite-difference time-domain method, or discrete dipole approximation, which are beyond the scope of the present GLMT-MST model and will be considered in future work.

### 3.3. Effect of Gold-Core Radius on N_z_

**Figure 8:**
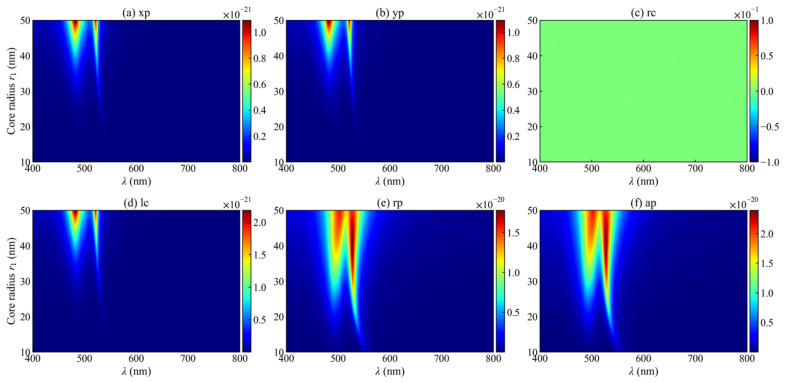
Size evolution of the axial optical torque of coated gold nanospheres with different gold-core radii. The calculation parameters are *l* = 1, α_0_ = 30°, and $\tau_{p}$ = 4.0 nm. The horizontal axis represents the incident wavelength, and the vertical axis represents the gold-core radius.

After analyzing the modulation of the torque spectra by the CABB shell thickness, we further investigate the effect of the gold-core radius on the intrinsic response of the core–shell particle. The gold-core radius not only changes the scattering cross section of the particle, but also modifies the size parameter and the excitable Mie resonance channels. Therefore, under fixed conditions of *l* = 1, *α*_0_ = 30°, and $\tau_{p}$ = 4.0 nm, Figure 8 presents the variation in *N_z_* with the incident wavelength and gold-core radius *r*_1_.

As shown in Figure 8, when the gold-core radius is small, the axial torque response remains relatively weak across all polarization states. As *r*_1_ increases, the strong-response regions gradually become enhanced, and distinct resonance branches appear in the wavelength range of approximately 480–550 nm. The response distributions under *xp*-, *yp*-, and *lc*-polarized incidence are relatively similar, with strong torques mainly appearing at larger gold-core radii. This indicates that the size-evolution behaviors under linear and *lc*-polarized incidence exhibit a certain degree of consistency. By contrast, under *rp*- and *ap*-polarized incidence, the torque magnitude increases to the order of 10^−20^ N·m, and the strong-response region becomes broader, indicating that radial and azimuthal polarizations can couple more efficiently to the strong-torque channels of larger core–shell particles. Under *rc*-polarized incidence, *N_z_* remains close to zero over the entire size and wavelength range, indicating that changing the gold-core radius alone cannot open the net axial angular-momentum transfer channel for this polarization state. These results show that the gold-core size not only affects the peak torque magnitude, but also modifies the position, width, and polarization dependence of the resonance branches, making it another structural degree of freedom for torque regulation in addition to the CABB shell thickness.

**Figure 9:**
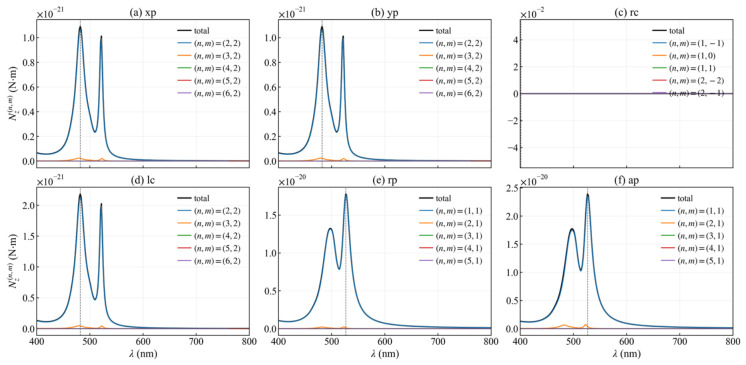
Contributions of different (*n*, *m*) channels to the axial optical torque *N_z_* spectra at representative peak radii selected from Figure 8. The calculation parameters are *l* = 1, *α*_0_ = 30°, and $\tau_{p}$ = 4.0 nm.

To further clarify the modal origin of the size evolution observed in Figure 8, Figure 9 presents an (*n*, *m*)-channel decomposition at representative strong-response points, with the calculation parameters set as *l* = 1, *α*_0_ = 30°, and $\tau_{p}$ = 4.0 nm. This decomposition is based on the series expressions of the axial torque in Equations (10) and (11). By extracting the contributions of different (*n*, *m*) channels to the *N_z_* spectra, the correspondence between these channel-resolved contributions and the total torque spectra can be compared. It should be emphasized that this decomposition is mainly used for modal diagnosis. Its purpose is not to simplify the actual scattering process into a single channel, but to identify the dominant multipolar–azimuthal channels responsible for axial angular-momentum transfer under different polarization states. As shown in Figure 9, for *xp*-, *yp*-, and *lc*-polarized incidence, the total torque spectra are almost dominated by the (*n*, *m*) = (2, 2) channel. The peak positions and spectral profiles of this channel are highly consistent with those of the total spectra, while the contributions from other (*n*, *m*) channels are relatively weak. The corresponding representative peak condition is *r*_1_ = 50.0 nm and λ = 481.9 nm. In contrast, under *rp*- and *ap*-polarized incidence, the total torque spectra are mainly determined by the contribution from the (*n*, *m*) = (1, 1) channel, with the representative peak condition of *r*_1_ = 41.0 nm and λ = 526.5 nm. Under *rc*-polarized incidence, the contributions from all (*n*, *m*) channels remain close to zero, consistent with the near-zero-torque response observed in Figure 8. In this case, *r*_1_ = 10.0 nm and λ = 401.0 nm are selected as the reference condition. These results indicate that the changes in the torque spectra induced by increasing the gold-core size cannot be simply interpreted as an increase in the number of higher-order modes. Instead, they should be understood as a selective coupling between specific (*n*, *m*) channels and the angular-momentum structure of the incident Bessel beam. Specifically, the gold-core radius mainly modifies the multipolar resonance channels of the core–shell particle that can be efficiently excited, whereas the polarization state of the incident light determines the coupling efficiency between these channels and the angular-momentum input of the optical field. Therefore, the gold-core size and beam polarization state select the dominant torque channels from the perspectives of the particle’s intrinsic response and the incident angular-momentum channels, respectively, and jointly determine the spectral profile, peak magnitude, and polarization dependence of *N_z_*.

However, a limitation concerning the large-core results should be emphasized. The representative conditions $r_{1}=41$ nm and $r_{1}=50$ nm lie in a size range where Lorentz-friction-induced plasmon damping and the associated nonharmonic renormalization of the effective dielectric response may become appreciable. Because these effects are not explicitly included in Equations (1) and (2), the peak wavelengths, linewidths, and absolute torque magnitudes reported for the largest gold cores should be regarded as model-dependent predictions within the classical local-response GLMT framework. Accordingly, the channel decompositions in Figure 9 are primarily intended to identify the dominant $\left(n,m\right)$ contributions within the adopted model. A quantitatively rigorous treatment of particles in this size range would require a self-consistent RPA-based dielectric response including Lorentz friction [68].

### 3.4. Effect of Incident Vector Bessel-Beam Parameters on N_z_

**Figure 10:**
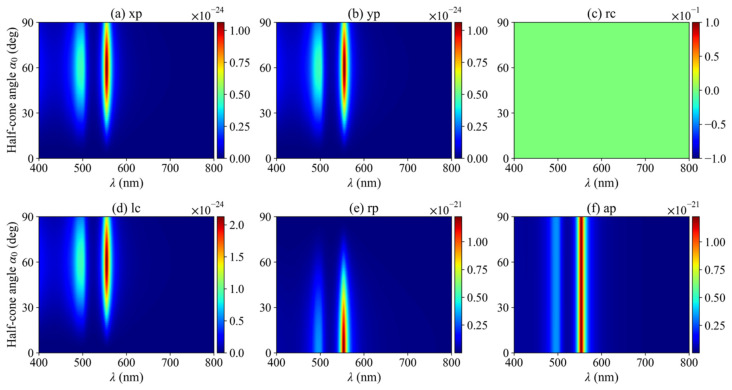
Two-dimensional distributions of the axial optical torque under different incident polarization states for a fixed coated structure. The calculation parameters are r_1_ = 10 nm, $\tau_{p}$ = 4.0 nm, and *l* = 1. The horizontal axis represents the incident wavelength λ, and the vertical axis represents the Bessel-beam half-cone angle α_0_.

After clarifying the effects of the CABB shell thickness and gold-core size on the intrinsic response of the coated particle, it is further necessary to investigate the modulation of *N_z_* by the parameters of the incident vector Bessel beam. Unlike structural parameters, the incident optical field does not change the material composition or geometry of the particle itself. Instead, it affects the coupling efficiency with the core–shell particle’s resonance channels by modifying the angular-momentum input, the composition of the field components, and the spatial distribution of the beam. Under the fixed conditions of *r*_1_ = 10 nm, $\tau_{p}$ = 4.0 nm, and *l* = 1, Figure 10 presents the two-dimensional distributions of *N_z_* as functions of the incident wavelength λ and half-cone angle *α*_0_ under different polarization states.

As shown in Figure 10, the torque distributions under *xp*- and *yp*-polarized incidence are nearly identical, with the main responses concentrated in two wavelength bands near 500 nm and 560 nm. This indicates that the axisymmetric coated sphere exhibits approximately equivalent coupling responses to the two orthogonal linear polarizations. Under *lc*-polarized incidence, the response wavelengths are generally close to those under linearly polarized incidence, but the torque magnitude increases to the order of 10^−24^ N·m, indicating that the spin angular momentum introduced by circular polarization can enhance axial angular-momentum transfer in the corresponding channels. In contrast, under *rp*- and *ap*-polarized incidence, the strong responses reach the order of 10^−21^ N.m, which is much higher than those under linear and circular polarizations. The strong-response regions are mainly distributed near the long-wavelength resonance band, suggesting that the vector-field structures of radial and azimuthal polarizations are more efficiently matched with the strong-torque channels of the coated particle. It is worth noting that the main response regions in Figure 10 mostly appear as nearly vertical wavelength bands; that is, the peak wavelength changes only weakly with α_0_, whereas the peak magnitude is more sensitive to α_0_. This indicates that the resonance wavelength is mainly determined by the intrinsic electromagnetic response of the gold-core–CABB-shell system, while the half-cone angle primarily modifies the coupling strength by adjusting the relative proportions of the field components and the vector Bessel beam’s beam shape coefficients (BSCs). Therefore, once the coated structure is fixed, the polarization state and half-cone angle can serve as external optical-field degrees of freedom for selectively exciting and modulating the intensity of existing torque-resonance channels.

**Figure 11:**
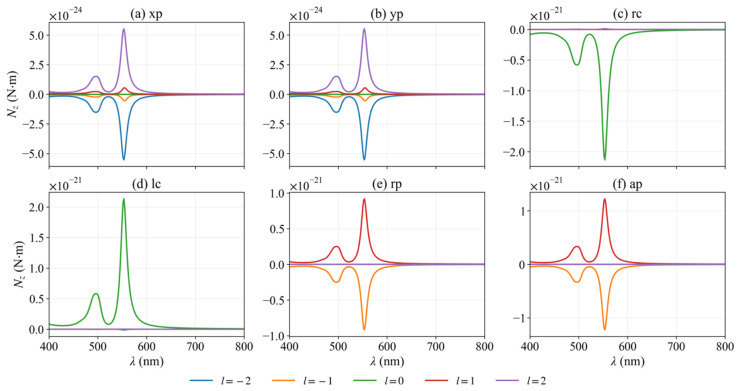
Representative axial optical torque spectra for different Bessel-beam orders *l*. The calculation parameters are *α*_0_ = 30°, r_1_ = 10 nm, and $\tau_{p}$ = 4.0 nm.

In addition to the polarization state and half-cone angle, the Bessel-beam order *l* further determines the helical phase structure of the incident field and the corresponding orbital angular-momentum channels. Figure 11 compares representative axial torque spectra for different values of *l* under fixed conditions of *α*_0_ *=* 30°, *r*_1_ = 10 nm, and $\tau_{p}$ = 4.0 nm. It can be seen that different polarization states exhibit pronounced selectivity with respect to *l*. For *xp*- and *yp*-polarized incidence, *l* = 2 and *l* = −2 generate the main torque peaks with opposite signs, while the peak positions are concentrated near similar resonance wavelengths. This indicates that changing the helical phase direction can reverse the direction of axial angular-momentum transfer in the linearly polarized channels. For *lc*- and *rc*-polarized incidence, the response is most pronounced at *l* = 0, and the two cases exhibit torque peaks with opposite signs. This indicates that, under circular polarization, the spin angular momentum of the incident light can drive a pronounced axial torque even without introducing orbital angular momentum. For *rp*- and *ap*-polarized incidence, the dominant responses are concentrated in the *l* = 1 and *l* = −1 channels, respectively, with positive and negative beam orders corresponding to torque peaks of opposite signs. This reflects the selective matching relationship between the vectorial polarization structure and the Bessel-beam order. Therefore, the direction and magnitude of *N_z_* are not determined solely by material absorption or particle resonance, but by the combined action of the scattering channels of the coated particle and the total angular-momentum channels of the incident structured light. The Bessel-beam order *l* can regulate not only the torque magnitude and spectral line shape, but, more importantly, the direction of the axial torque without changing the particle structure. This provides a direct external degree of freedom for reversible rotational control of structured-light-driven nanorotors.

Overall, the axial optical torque of CABB-coated gold nanospheres is jointly determined by the particle structure and the incident optical field. The shell thickness mainly regulates the torque-resonance wavelength and spectral line shape, while the gold-core radius modifies the excitable Mie resonance channels. The polarization state determines the coupling efficiency of different channels, whereas the Bessel-beam order *l* provides an external degree of freedom for torque-direction reversal. These results indicate that a selective matching relationship exists between the CABB-shell-modulated core–shell Mie response and the angular-momentum input of the vector Bessel beam, providing a theoretical basis for the design of tunable optical nanorotors.

Before closing this section, the experimental feasibility of the proposed system should be briefly discussed. For the material structure, gold nanospheres with radii ranging from several to tens of nanometers can be routinely synthesized by colloidal methods with good size control [69]. Meanwhile, Cs_2_AgBiBr_6_ has been experimentally prepared in the form of thin films, nanocrystals, nanosheets, and microcrystals, indicating that this lead-free double perovskite can be processed into nanoscale structures. In particular, colloidal Cs_2_AgBiBr_6_ nanocrystals have been synthesized and purified for solution-processed optoelectronic applications, and Cs_2_AgBiBr_6_ nanosheets with thicknesses of several nanometers have also been reported [70]. These studies suggest that forming a thin perovskite coating layer a few nanometers thick is experimentally feasible. In practice, Cs_2_AgBiBr_6_-coated gold nanospheres may be attempted through seed-mediated growth, ligand-assisted deposition, or heterogeneous nucleation of perovskite precursors on preformed Au nanoparticles [53]. However, achieving perfectly concentric and atomically smooth Cs_2_AgBiBr_6_ shells remains challenging. Therefore, the present model should be regarded as an idealized theoretical structure that captures the fundamental electromagnetic mechanism of shell-mediated optical torque modulation.

Generating the required vector Bessel beams is also experimentally feasible. Scalar and higher-order Bessel beams can be generated using axicons, computer-generated holograms, or spatial light modulators [71]. Vector Bessel beams with radial or azimuthal polarization can be obtained by combining Bessel-beam generation methods with polarization converters, q-plates, or spatially structured polarization modulation [72]. Previous experimental studies have demonstrated radially and azimuthally polarized Bessel beams and vector vortex beams using such optical elements [73]. Therefore, the polarization state, Bessel-beam order, and half-cone angle considered in this work correspond to experimentally controllable optical parameters. These considerations indicate that although the present work is theoretical, the proposed Cs_2_AgBiBr_6_-coated Au nanosphere illuminated by vector Bessel beams is experimentally relevant and may provide guidance for future structured-light-driven rotational-manipulation experiments.

## 4. Conclusions

In this study, the axial optical torque *N_z_* exerted on CABB perovskite-coated gold nanospheres under vector Bessel-beam illumination is investigated based on GLMT and the MST method. It is found that the CABB shell does not simply increase the outer radius of the particle, but introduces material dispersion and the core–shell interfacial response into the angular-momentum exchange process between the gold core and the structured optical field. Compared with bare gold structures, the CABB-coated particle exhibits pronounced changes in the torque peak position and spectral line shape. As the shell thickness varies, the torque spectra further show continuous spectral shifts and peak-shape reconstruction. These results indicate that the CABB shell is not merely a geometrical coating layer, but an effective structural degree of freedom for regulating the axial angular-momentum response of core–shell particles.

Analyses of the near-field distributions, energy-flow distributions, and modal (*n*, *m*) channels show that a strong axial torque is not simply determined by maximum local field intensity. Field enhancement and energy-flow redistribution near the core–shell interface indicate enhanced local coupling between the particle and the incident light. However, the formation of a net *N_z_* also requires angular-momentum channel matching. In other words, only when the multipolar scattering channels of the core–shell particle are efficiently coupled with the spin and orbital angular-momentum components and the polarization structure of the vector Bessel beam can the local electromagnetic response be converted into a pronounced axial optical torque. This result also explains why different polarization states yield dramatically different torque magnitudes, spectral line shapes, and even near-zero responses.

In summary, the *N_z_* modulation strategy proposed in this study does not rely solely on enhancing the local field. Instead, it simultaneously controls both particle-related and optical-field-related angular-momentum channels. The intrinsic scattering response of the core–shell particle can be regulated by tuning the CABB shell thickness and gold-core size, while the angular-momentum input of the incident optical field can be adjusted through the polarization state and Bessel-beam order *l*. By combining these two types of degrees of freedom, the working wavelength, response magnitude, and rotational direction of the optical torque can be effectively regulated. These results may be useful for the design of tunable optical nanorotors driven by structured light and may also provide guidance for reversible rotational manipulation of nanoparticles, polarization-selective micro-/nanomanipulation, and perovskite–plasmonic hybrid optomechanical devices.

However, it should be noted that the present results are obtained within a classical local-response description of the gold core. For gold-core radii of several tens of nanometers, additional nanoscale plasmon damping and dielectric-function renormalization associated with Lorentz friction may quantitatively alter the predicted resonance positions, linewidths, and optical torque magnitudes. Therefore, the results obtained for larger gold cores should be interpreted within the limitations of the adopted Drude–Sommerfeld model. Extending the present GLMT–MST framework by incorporating an effective dielectric response constitutes an important direction for future work.

## Data Availability

The data that support the findings of this study are available from the corresponding author upon reasonable request.

## Appendix A

For polarized Bessel beams, the corresponding beam shape coefficients (BSCs) were derived in our previous work. For convenience, they are summarized here.

(A1) $$\left\{\begin{array}{l} g_{n,\mathrm{TM}}^{m,u}=\left\{\begin{array}{cc} \frac{n(n+1)}{i^{n+1}(2n+1)}q_{mn}^{u} & m\geq 0\\ \frac{1}{i^{n+1}{\left(-1\right)}^{m}}\frac{(n+m)!}{(n-m)!}\frac{n(n+1)}{\left(2n+1\right)}q_{mn}^{u} & m<0 \end{array}\right.\\ g_{n,\mathrm{TE}}^{m,u}=\left\{\begin{array}{cc} \frac{-in(n+1)}{i^{n+1}(2n+1)}p_{mn}^{u} & m\geq 0\\ \frac{-i}{i^{n+1}{\left(-1\right)}^{m}}\frac{(n+m)!}{(n-m)!}\frac{n(n+1)}{\left(2n+1\right)}p_{mn}^{u} & m<0 \end{array}\right. \end{array}\right.$$

where

(A2) $$\left\{\begin{array}{l} p_{mn}^{u}=-4i^{n+1}D_{mn}e^{-ik_{z}z_{0}}\left[\pi_{mn}\left(\cos\alpha_{0}\right)I_{+}^{u}+\tau_{mn}\left(\cos\alpha_{0}\right)I_{-}^{u}\right]/4\pi^{2}\\ q_{mn}^{u}=-4i^{n+1}D_{mn}e^{-ik_{z}z_{0}}\left[\tau_{mn}\left(\cos\alpha_{0}\right)I_{+}^{u}+\pi_{mn}\left(\cos\alpha_{0}\right)I_{-}^{u}\right]/4\pi^{2} \end{array}\right.$$

Here, $D_{mn}=\frac{(2n+1)(n-m)!}{4n(n+1)(n+m)!}$, $\pi_{mn}(\cos\theta )=m\frac{P_{n}^{m}(\cos\theta )}{\sin\theta }$, $\tau_{mn}(\cos\theta )=\frac{dP_{n}^{m}(\cos\theta )}{d\theta }$, $P_{n}^{m}(x)={\left(-1\right)}^{m}{\left(1-{\left(\cos\theta \right)}^{2}\right)}^{m/2}\frac{d^{m}}{dx^{m}}P_{n}(\cos\theta )$.

For *xp*-linear polarization:

(A3) $$I_{\pm }^{xp}=\pi \left[i^{1+l-m}e^{i(1+l-m)\phi_{0}}J_{1+l-m}\left(\sigma_{0}\right)\right.\left.\pm i^{-1+l-m}e^{i(-1+l-m)\phi_{0}}J_{-1+l-m}\left(\sigma_{0}\right)\right]$$

where $\phi_{0}=\arctan\left(y_{0}/x_{0}\right)$, $\sigma_{0}=-k\rho_{0}\sin\alpha_{0}$, $\rho_{0}=\sqrt{x_{0}^{2}+y_{0}^{2}}$.

For *yp*-linear polarization:

(A4) $$I_{\pm }^{yp}=-i\pi \left[i^{1+l-m}e^{i(1+l-m)\phi_{0}}J_{1+l-m}\left(\sigma_{0}\right)\right.\left.\mp i^{-1+l-m}e^{i(-1+l-m)\phi_{0}}J_{-1+l-m}\left(\sigma_{0}\right)\right]$$

For circular polarization:

(A5) $$\left\{\begin{array}{c} p_{mn}^{cp}\\ q_{mn}^{cp} \end{array}\right\}=\frac{1}{\sqrt{2}}\left\{\begin{array}{c} p_{mn}^{xp}\\ q_{mn}^{xp} \end{array}\right\}\pm \frac{i}{\sqrt{2}}\left\{\begin{array}{c} p_{mn}^{yp}\\ q_{mn}^{yp} \end{array}\right\}$$

For radial polarization:

(A6) $$\left\{\begin{array}{l} I_{+}^{rp}=2\pi i^{l-m}e^{i(l-m)\phi_{0}}J_{l-m}\left(\sigma_{0}\right)\\ I_{-}^{rp}=0 \end{array}\right.$$

For azimuthal polarization:

(A7) $$\left\{\begin{array}{l} I_{+}^{ap}=0\\ I_{-}^{ap}=-i2\pi i^{l-m}e^{i(l-m)\phi_{0}}J_{l-m}\left(\sigma_{0}\right) \end{array}\right.$$

## Appendix B

The analytical expressions for the scattering-field coefficients of a concentric core–shell sphere were derived in our previous work and are summarized here for ease of reference.

(A8) $$A_{n}^{m,u}=a_{n}g_{n,TM}^{m,u},B_{n}^{m,u}=b_{n}g_{n,TE}^{m,u}$$

Here, $a_{n}$ and $b_{n}$ denote the conventional Mie scattering coefficients of the coated sphere, whose explicit expressions are given below:

(A9) $$\begin{array}{l} a_{n}=\frac{\psi_{n}(y)\left[\psi_{n}^{\prime }\left(m_{2}y\right)-A_{n}\chi_{n}^{\prime }\left(m_{2}y\right)\right]-m_{2}\psi_{n}^{\prime }(y)\left[\psi_{n}\left(m_{2}y\right)-A_{n}\chi_{n}\left(m_{2}y\right)\right]}{\xi_{n}(y)\left[\psi_{n}^{\prime }\left(m_{2}y\right)-A_{n}\chi_{n}^{\prime }\left(m_{2}y\right)\right]-m_{2}\xi_{n}^{\prime }(y)\left[\psi_{n}\left(m_{2}y\right)-A_{n}\chi_{n}\left(m_{2}y\right)\right]}\\ b_{n}=\frac{m_{2}\psi_{n}(y)\left[\psi_{n}^{\prime }\left(m_{2}y\right)-B_{n}\chi_{n}^{\prime }\left(m_{2}y\right)\right]-\psi_{n}^{\prime }(y)\left[\psi_{n}\left(m_{2}y\right)-B_{n}\chi_{n}\left(m_{2}y\right)\right]}{m_{2}\xi_{n}(y)\left[\psi_{n}^{\prime }\left(m_{2}y\right)-B_{n}\chi_{n}^{\prime }\left(m_{2}y\right)\right]-\xi_{n}^{\prime }(y)\left[\psi_{n}\left(m_{2}y\right)-B_{n}\chi_{n}\left(m_{2}y\right)\right]} \end{array}$$

where $A_{n}$ and $B_{n}$ are expressed as follows:

(A10) $$\begin{array}{l} A_{n}=\frac{m_{2}\psi_{n}\left(m_{2}x\right)\psi_{n}^{\prime }\left(m_{1}x\right)-m_{1}\psi_{n}^{\prime }\left(m_{2}x\right)\psi_{n}\left(m_{1}x\right)}{m_{2}\chi_{n}\left({\tilde{n}}_{2}x\right)\psi_{n}^{\prime }\left(m_{1}x\right)-m_{1}\chi_{n}^{\prime }\left(m_{2}x\right)\psi_{n}\left(m_{1}x\right)}\\ B_{n}=\frac{m_{2}\psi_{n}\left(m_{1}x\right)\psi_{n}^{\prime }\left(m_{2}x\right)-m_{1}\psi_{n}^{\prime }\left(m_{1}x\right)\psi_{n}\left(m_{2}x\right)}{m_{2}\chi_{n}^{\prime }\left(m_{2}x\right)\psi_{n}\left(m_{1}x\right)-m_{1}\chi_{n}\left(m_{2}x\right)\psi_{n}^{\prime }\left(m_{1}x\right)} \end{array}$$

The auxiliary functions appearing in Equation (A10) are defined as follows:

(A11) $$\begin{array}{l} \psi_{n}(x)=xj_{n}(x),\chi_{n}(x)=-xy_{n}(x),\xi_{n}(x)=xh_{n}^{\left(1\right)}(x)\\ x=kr_{1},y=kr_{2} \end{array}$$
